# Chlorambucil-Bearing Hybrid Molecules in the Development of Potential Anticancer Agents

**DOI:** 10.3390/molecules28196889

**Published:** 2023-09-30

**Authors:** Sijongesonke Peter, Blessing Atim Aderibigbe

**Affiliations:** Department of Chemistry, University of Fort Hare, Alice 5700, South Africa; 201414787@ufh.ac.za

**Keywords:** cancer, chlorambucil, drug resistance, hybridization, treatment

## Abstract

Increasing cases of cancer have been a primary concern in recent decades. Developing new chemotherapeutics is challenging and has been faced with limitations, such as multidrug resistance, poor specificity, selectivity, and toxicity. The aforementioned factors contribute to treatment failure. Hybrid compounds have features that can overcome the limitations mentioned above. Chlorambucil, an anticancer drug that is used to treat prostate and breast cancer, suffers from poor aqueous solubility and specificity, a short half-life, and severe side effects, including anaemia and bone marrow suppression. It compromises the immune system, resulting in treatment failure. Hence, its combination with other pharmacophores has been reported to result in effective anticancer agents with fewer side effects and high therapeutic outcomes. Furthermore, this review gives an update (2010 to date) on the developments of chlorambucil hybrid compounds with anticancer activity, and the structure-activity relationship (SAR), and also highlights future strategies for developing novel anticancer agents.

## 1. Introduction

The increasing number of cases of cancer worldwide is a major health crisis [1,2,3,4]. Cancer is one of the leading causes of death. Notably, it is caused by the unlimited growth of abnormal cells in body organs, sometimes resulting in damage to other organs if it is not detected and treated early, resulting in metastatic cancer (advanced stages) [1,2,3,4,5,6]. It has been widely reported that an individual’s lifestyle, such as alcohol intake, obesity, lack of exercise, and smoking, are factors that contribute to cancer. However, inherited genes (5–10%) and gene mutations (90–95%) are major causes of cancer globally [1,2,3,4,5,6]. Cancer types are classified according to the body organ they affect [6]. A concerning challenge is that cancer-related deaths are high in Africa and Asia [6,7,8,9]. Hence, there is a pressing need for the development of effective and affordable chemotherapeutics.

Various strategies are used to treat cancer, including surgical methods, radiography, and chemotherapy. Chemotherapy is widely recognized as a better, safer, and more effective approach. Currently, it is the most commonly used approach [10]. Anticancer agents suffer from several limitations, such as poor solubility, specificity, selectivity, multidrug resistance, instability, and toxicity [10,11]. Thus, their drawbacks contribute to increasing cancer deaths [2,5]. Additionally, drug discovery failures also contribute to a delay in the development of new anticancer drugs [12]. Among the compounds used to treat cancer is chlorambucil, an alkylating anticancer agent which has been used to treat breast, ovary, testicular cancer, and leukaemia for several decades [12]. This therapeutic agent binds to the DNA and hinders replication, resulting in cell death [12].

**Figure 1:**
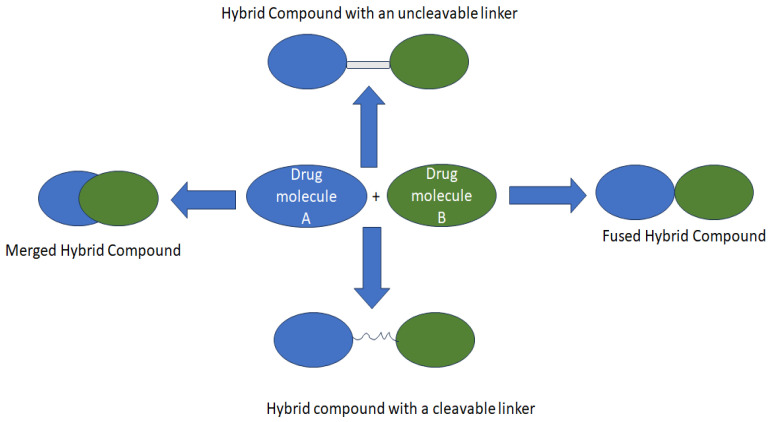
Strategies used to design hybrid drugs.

Nonetheless, its unknown optimal dose and non-specificity result in toxicity, leading to some severe side effects which compromise its anticancer activity [10,13,14]. Consequently, hybridizing anticancer compounds through drug repurposing/repositioning is one of the best strategies to overcome these limitations (Figure 1) [15,16,17]. Therefore, modifying Chlorambucil through the development of its hybrid derivatives has been investigated and is still an ongoing strategy to improve its efficacy. This review reports currently developed Chlorambucil hybrid compounds.

## 2. Cancer Update

The rapidly increasing numbers of cancer cases and deaths show that current cancer treatments have limitations with numerous drawbacks. Additionally, these rising numbers are affecting the public healthcare system financially, with a lot of funds spent on anticancer drug discovery, surgeries, and hospitalization [18,19,20,21]. The common cancers recorded in the United States are those of the breast, colon, prostate, skin, kidney, liver, pancreatic, and renal pelvis. Furthermore, lung, prostate, breast, and colorectal cancer are regarded as the most common and deadly cancers around the globe among men and females [21].

An estimation from different researchers suggests that the number of cancer cases could double in most countries between 2030 and 2040 [8,20,21,22,23]. The International Agency for Research on Cancer reported more than 18 million cancer cases and 9.6 million cancer-related deaths in 2018 [22]. Reports from different sources (GLOBOCAN and GCO) estimated an increasing number of cancer cases with 19 million in 2020, with the number of cancer-related deaths reaching 9.6 million in the same year. Thus, there is a pressing need to develop new and effective chemotherapeutics for the treatment of cancer.

## 3. Hybrid Drug Strategy for the Treatment of Cancer

Hybrid drugs are a combination of two or more pharmacophores, resulting in a single-entity drug with dual targets [16,24,25]. Treating cancer with single-entity drugs suffers from several limitations, such as lack of selectivity and drug resistance, due to the complexity of cancers and their mechanism of resistance [16,24,25]. Hence, hybrid drugs are designed to combat these limitations. Some hybrid drugs do not obey Lipinski’s rule. However, several researchers have reported some of the advantages of hybrid compounds, such as interaction with several targets and reduced drug–drug interaction, among other strategies such as fixed-dose therapy [16,24,25].

Additionally, combining already known pharmacophores into a single molecule with known pharmacokinetics and pharmacodynamics results in drugs with reduced toxicity and side effects. Even their development consumes less time. Hence, several researchers are exploring this strategy [16,24,25]. In the development of anticancer drugs, hybrid drugs have been widely explored using different synthetic approaches [24,25]. The combination of two pharmacophoric moieties with the same or different mode of action, using functional groups directly or indirectly (linkers or a spacer between the pharmacophores) has been widely investigated. The linkers or spacers are categorized as non-cleavable and cleavable. Cleavable linkers mostly result in hybrid prodrugs, such as amides, esters, and carbamates that release the parent drugs to the target biological site. On the contrary, non-cleavable linkers result in hybrid drugs and sustain the hybrid structure in enzymatic conditions throughout the action process [24,25]. Thus, Chlorambucil-based hybrid compounds with different pharmacophores have been synthesized to improve their specificity, reduce toxicity, and eradicate side effects.

## 4. About Chlorambucil

**Figure 2:**
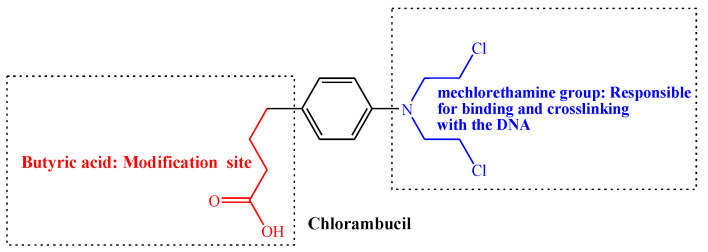
Structure of Chlorambucil.

Chlorambucil is an alkylating agent used in cancer therapy (Figure 2). It belongs to the aryl nitrogen mustard class of drugs, which was developed by Alexander Haddow in the early 1950s at the Chester Beatty Institute, currently known as “The Institute of Cancer Research” [26]. They published their first report on Chlorambucil in 1955 on its biological activity on malignant lymphoma. Thus, Chlorambucil together with melphalan and busulphan are the first treatment for chronic lymphocytic leukaemia, myeloma, and myeloid leukaemia [13,26,27]. The Food and Drug Administration (FDA) approved the clinical use of Chlorambucil to treat chronic lymphocytic leukaemia in 2008. It was also approved to treat patients with indolent B-cell non-Hodgkin lymphoma [2,28].

Moreover, it is typically used to treat Hodgkin’s disease, chronic lymphosarcoma, lymphocytic leukaemia, giant follicular lymphoma, and malignant lymphoma [28,29]. Although it is a potent drug to treat different cancer types, its anticancer activity is hindered by numerous drawbacks, such as a lack of specificity, drug resistance, toxicity, and a short half-life. Therefore, its modification is an attractive approach for developing effective anticancer drugs [19,28,29,30].

Generally, nitrogen mustard drugs are alkylating agents with the bis(2-chloroethyl) group that acts as a chemical constituent. They are also genotoxic and mutagenic to the cancer cells [28,31]. These chemotherapeutic agents, including Chlorambucil, prevent cell proliferation and DNA replication through cross-linking and binding the DNA of cancer cells [10,19]. Additionally, Chlorambucil uses the bis(2-chloroethyl) group to bind the nucleobases guanine (at N7) and adenine (at N3), resulting in the prevention of DNA replication and damage via DNA strand linking. Consequently, the formation of covalent bonds leads to the activity inhibition of corresponding biomacromolecules. However, they are toxic to normal cells since the construction of the covalent bond occurs in both cancer and normal cells [2,5,10,19,30,31]. Nevertheless, poor selectivity and high toxicity result in Chlorambucil chemoresistance [28]. Thus, there is a pressing need to modify Chlorambucil via the butyric acid group to improve its activity and reduce its toxicity to normal cells.

## 5. Chlorambucil-Based Hybrid Compounds with Anticancer Activity

### 5.1. Chlorambucil–Estradiol Hybrids

**Figure 3:**
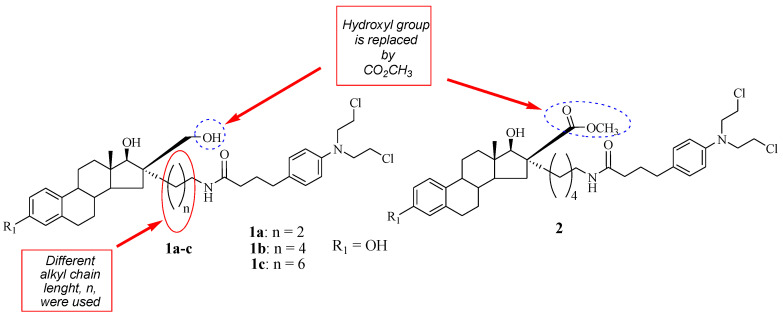
Chemical structure of Chlorambucil–estradiol hybrids substituted at the 16 α (**1a**–**c**) and 16 β positions (**2**) [31].

A class of Chlorambucil–estradiol compounds was synthesized by Gupta et al. as a potential treatment for breast cancer [31]. A series of compounds were synthesized through the introduction of Chlorambucil at position 16α (compounds **1a**–**c**) and 16β (compound **2**) of the estrone moiety, and the desired compounds were evaluated for anti-breast cancer activity against MCF-7 (hormone-dependent) and hormone-independent (MDA-MB-436 and MDA-MB-486) cancer cell lines (Figure 3). These compounds exhibited moderate cytotoxic activity influenced by their chain length and concentration in vitro [31]. Additionally, SAR indicated that increasing the chain length compromised the cytotoxic effect of the compounds. Compound **1a** (IC_50_ = 40 µM) with a short chain length exhibited a superior cytotoxic effect than its counterparts against hormone-independent cancer cell lines. However, the trend was the opposite against the hormone-dependent cancer cell lines [31]. Moreover, it was noted that compound **2** with the −CO_2_ CH_3_ functional group at the 16 β position displayed no anticancer effect against all breast cancer cell lines used in the study. Modifying the hydroxymethyl group in position 16β of the estrone moiety results in biologically inactive compounds in vitro. Descôteaux et al. reported similar findings regarding the modification of hydroxymethyl groups [31,32]. However, further elucidation of these compounds in vivo is paramount.

### 5.2. Chlorambucil–Tyrosine Hybrids

**Figure 4:**
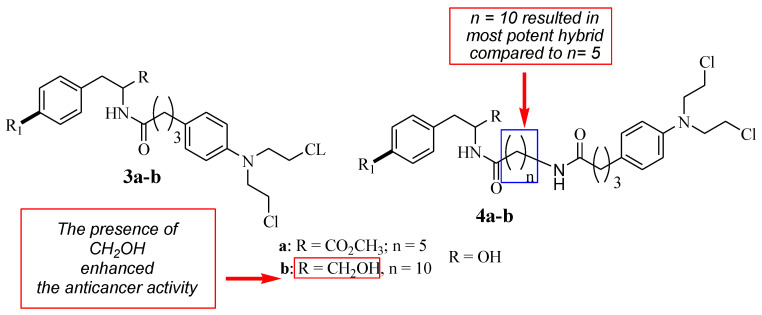
Chemical structures of the 1st generation of Chlorambucil–tyrosine hybrids (**3**–**4**) [32].

Descôteaux et al. reported Chlorambucil combined with D- and L-tyrosine analogues with anticancer activity [32]. They synthesized a series of hybrid compounds (**3**–**4**) and evaluated their anticancer activity against breast cancer cell lines (MCF-7 and MDA-MB-231) in vitro (Figure 4). The hybrid’s anticancer activity was more enhanced in comparison with Chlorambucil (IC_50_ > 130 µM for all cell lines). Specifically, L-hybrids (IC_50_ values between 19.39 and 67.90 µM) were generally superior to D-hybrids (IC_50_ values between 16.27 and 152.37 µM) [32]. Something noteworthy is that **L-4b** (IC_50_ = 19.39 µM) and **D-4b** (IC_50_ = 16.27 µM) were the most potent compounds against MCF-7. However, these two compounds displayed no significant effect against the MDA-MB-231 breast cancer cell line in vitro [32].

**Figure 5:**
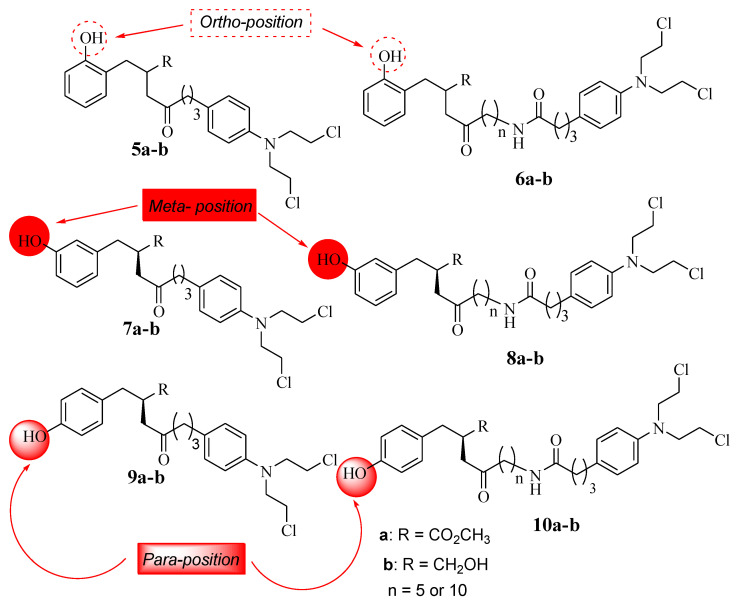
Chemical structures of the 2nd generation of Chlorambucil–tyrosine hybrids (**5**–**10**) [30].

The SAR studies showed that the anticancer effects of **L-4b** and **D-4b** were influenced by their long chain length (10 carbons) between the parental molecules (Chlorambucil and tyrosine) [30]. In addition, the increasing solubility due to the presence of CH_2_ OH also contributed to the improved anticancer effect, as the hydroxymethyl group is beneficial for biological potency [32]. Notably, different chemical methods (linear and convergent synthetic methods) were used to synthesize compounds **4a** and **4b**, and that influenced the yields of compounds synthesized via fewer steps, resulting in high yields and vice versa [32]. Brasseur and Descôteaux investigated a series of Chlorambucil–tyrosine hybrid molecules using the synthetic routes reported by Descôteaux et al. (2010) [31], and compounds **5**–**10** were evaluated against breast cancer cell lines (targeting estrogen receptor alpha) (Figure 5) [30]. The location of the phenol hydroxyl group was essential in the development of novel Chlorambucil–tyrosine hybrid drugs. Thus, meta-, ortho-, and para-substitutions were explored during the synthesis of these analogues by Brasseur and Descôteaux [30].

The IC_50_ values of the synthesized compounds (**5**–**10**) were in the range of 17.72–63.03 µM for meta-substituted hybrids **7** and **8**, 20.54–79.37 µM for ortho-substituted hybrids **5** and **6**, and 19.39–55.09 µM for para-substituted compounds **9** and **10**, with parent drugs displaying IC_50_ values of 130.36 µM for Chlorambucil and 136.8 µM for tyrosine against the MCF-7 and MDA-MB-231 cancer cell lines, respectively. Moreover, these compounds showed more specificity to hormone-dependent cancer cells, and the position of the phenol hydroxyl group (OH-*) influenced the activity of these analogues [30].

**Figure 6:**
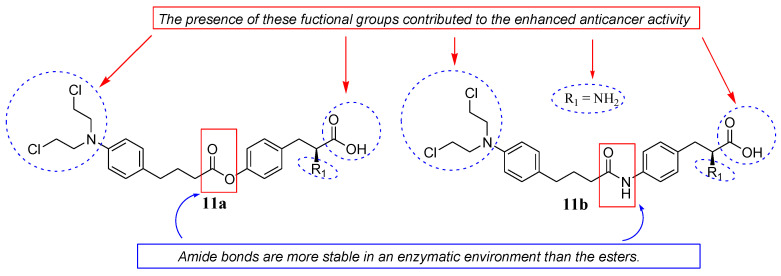
Chemical structures of Chlorambucil–tyrosine hybrids synthesized by Pocasap et al. via esterification (**11a**) and amidation (**11b**) [33].

Pocasap et al. synthesized a class of Chlorambucil–tyrosine hybrid compounds (**11a** and **11b**) by combining Chlorambucil with L-tyrosine via esterification and amidation reactions (Figure 6) [33]. The antiproliferative activity of these compounds was evaluated on MCF-7 breast cancer cell lines [33]. Their antiproliferative activity was time–concentration-dependent, with compounds **11a** and **11b** exhibiting higher antiproliferative activity and cell viability than Chlorambucil [33]. The presence of a free carboxylic and amino group, an ester/amide linkage, and an aromatic side chain were responsible for the improved chemotherapeutic effect of the compounds [33]. Additionally, the hybrid **11b** with an amide bond between the two drug scaffolds was preferable to compound **11a** with an ester bond because amide bonds are more stable in an enzymatic environment than esters. Animal studies for these two hybrids are recommended to further validate the findings [33].

### 5.3. Chlorambucil–Methionine Hybrid

**Figure 7:**
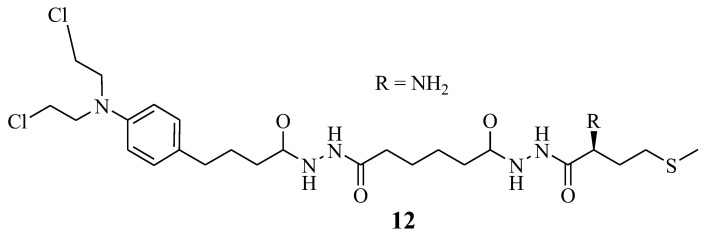
Chlorambucil–methionine Hybrid **12** [14].

Omoomi et al. synthesized Chlorambucil–methionine hybrid **12** to improve Chlorambucil efficacy and reduce its side effects (Figure 7) [14]. The MCF-7 breast cancer cell line was used to evaluate the anticancer effect of the novel compound. The in vitro biological studies of compound **12** showed that it exhibited a similar anticancer effect as Chlorambucil. However, it was less toxic than Chlorambucil. It is worth noting that this hybrid’s compound mode of action was via inducing apoptosis. However, further studies to validate these findings are desired [14].

### 5.4. Chlorambucil–7α-Testosterone Hybrid

**Figure 8:**
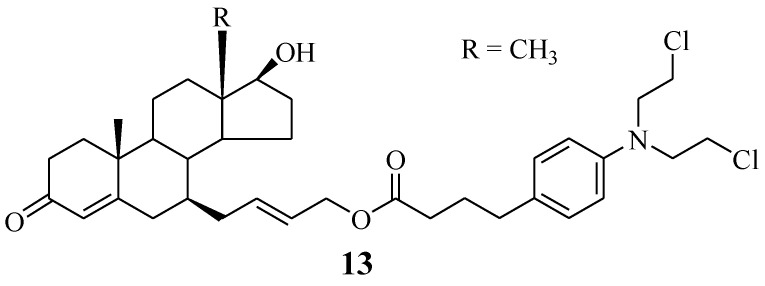
Chemical structure of Chlorambucil–7α-testosterone hybrid **13** [34].

A Chlorambucil–7α-testosterone hybrid compound **13** was reported by Bastien et al. (Figure 8). This compound was synthesized using two different synthetic approaches to improve its poor yield [32,34]. It was prepared through an SN2-type substitution reaction (six-step reaction) and an olefin cross-metathesis reaction (five-step reaction). Furthermore, the hybrid was evaluated against two prostate cancer cell lines (LNCaP and PC3) in vitro [34]. The in vitro findings indicated that this novel chemotherapeutic agent was selective towards cancer cells, with an IC_50_ value of 101.0 µM against LNCaP cancer cell lines, but inactive against PC3 cancer cell lines. Therefore, its good specificity makes it a promising hybrid molecule for the treatment of prostate cancer. However, further investigations are also needed. Something noteworthy is that fewer reaction steps resulted in higher yield and vice versa [34].

### 5.5. Chlorambucil–Platinum Hybrids

**Figure 9:**
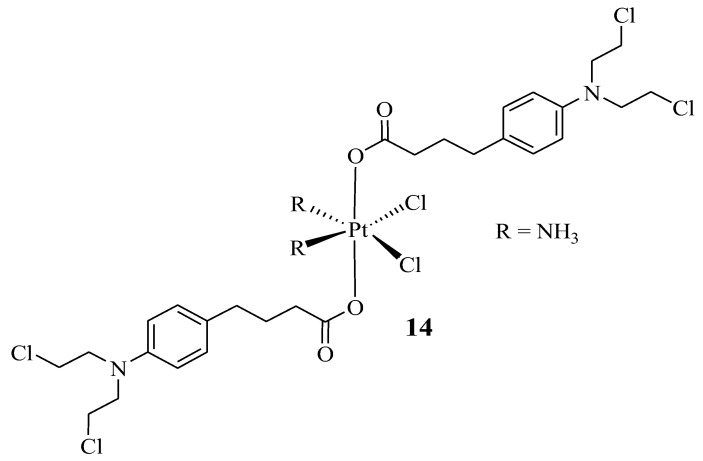
Chemical structure of Chlorambucil–platinum hybrid compound **14** [35].

Pathak et al. prepared a hybrid compound containing cisplatin with two moieties of Chlorambucil to enhance the anticancer efficacy of both drugs and reduce their severe side effects [35]. The synthesized hybrid **14** (Figure 9) was evaluated in vitro for its anticancer effect against several human cancer cells (breast (MCF-7), prostate (PC3), and ovarian (A2780) cancer cell lines), cisplatin-resistant human ovarian cancer cells (A2780/CP70), and mouse breast cancer cells (4T1) [35]. The addition of nanoparticles after the synthesis of the chlorambucil–cisplatin hybrid was considered due to the previous study by Pathak et al., where the addition of mitochondrial-targeted nanoparticles (NPs) resulted in the better delivery of drugs due to the ability of nanocarriers to improve loaded drug biodistribution, pharmacokinetics, and stability properties [35,36]. The findings from this study were consistent with the previous research by Pathak et al., as hybrid drug **14** displayed enhanced anticancer effects against PC3 and MCF-7 cancer cell lines than the parent molecules, cisplatin and Chlorambucil [35]. Furthermore, the encapsulation of NPs resulted in a significantly active chemotherapeutic agent, especially against prostate (PC3) and cisplatin-resistant human ovarian cancer cell lines, as shown in Table 1 [35].

**Figure 10:**
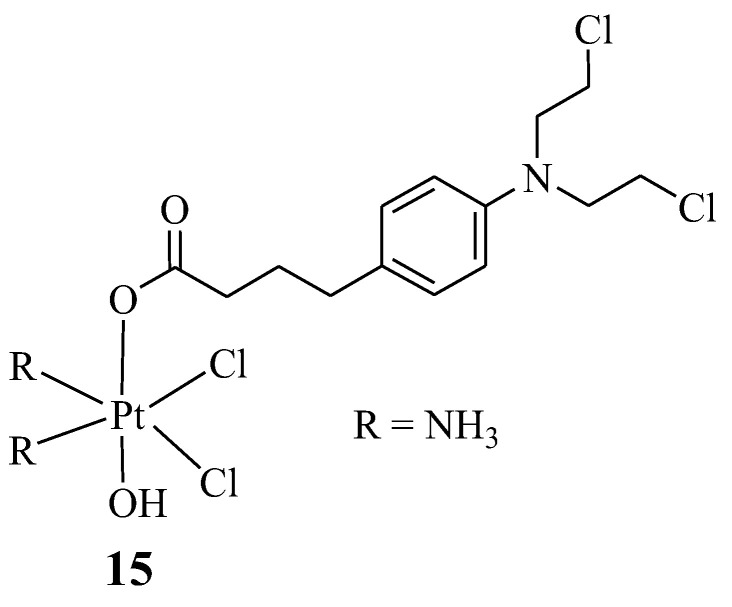
Chemical structure of Chlorambucil–platinum hybrid compound **15** [37].

Montagner et al. synthesized Chlorambucil–platinum hybrid compound **15** with one Chlorambucil moiety, compared to the two moieties synthesized by Pathak et al., (Figure 10) and evaluated the hybrid drug against several human cancer cell lines (C13*, HCT-15, 2008, BCPAP, PSN1, LoVo, and A431), in vitro [37]. As shown in Table 2, compound **15** exhibited superior cytotoxic activity on all the human cancer cell lines, even against drug-resistant strains, compared to the parent drugs. Additionally, its drug specificity (DNA-targeting ability) was higher than that of its parental drugs, resulting from its enhanced lipophilicity, which led to its high capability of crossing the cell membrane and higher accumulation in the tumor cells. Compound **15** is a promising chemotherapeutic agent with a high potential to overcome cisplatin resistance. This compound was also ten times more cytotoxic than other previously reported Chlorambucil–platinum hybrid molecules. Notably, this study revealed that combining two DNA-binding molecules to form a hybrid compound can further enhance their antitumor activity [37].

**Figure 11:**
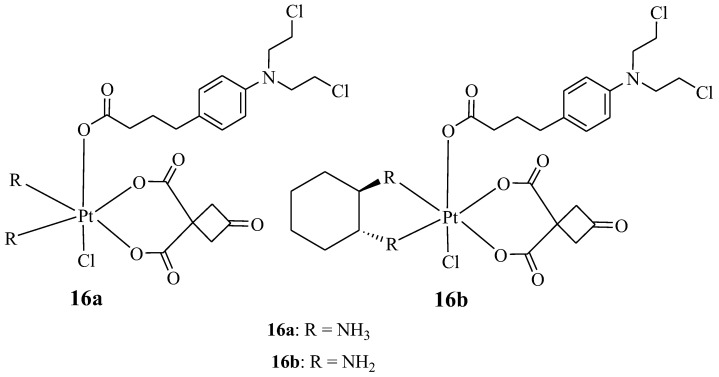
Chemical structures of platinum-based Chlorambucil hybrids with anticancer activity (**16a**,**b**) [38].

New platinum-based Chlorambucil hybrid compounds (**16a**,**b**) were synthesized by Chen et al. to improve the anticancer effect of Chlorambucil (Figure 11). The two obtained hybrid compounds were evaluated for their anticancer activity against several human cancer cell lines (both drug-sensitive and -resistant strains of cisplatin) in vitro [38]. The cancer cell lines used for the study included lung (A549/CDDP and A549), gastric (SGC-7901 and SGC-7901/CDDP), and human normal (HUVEC) cancer cell lines. Cisplatin and Chlorambucil were used as reference compounds [38]. It was noteworthy that compound **16a** displayed a high cytotoxic effect with IC_50_ values which were 62.04 and 34.93-fold lower than Chlorambucil against drug-resistant strains, whereas its activity was comparable to cisplatin when tested against drug-sensitive strains of cancer cell lines [38]. Additionally, hybrid **16a** reduced the drug resistance factor of cisplatin from 9.59 (cisplatin) to 0.58 and from 7.09 (cisplatin) to 0.81 against A549/CDDP and SGC-7901/CDDP, respectively. Therefore, this compound had the potential to overcome cisplatin drug resistance [38]. However, the anticancer activity of compound **16b** was comparable to cisplatin against all the cancer cell lines used. Compounds **16a** and **16b** were less toxic towards human normal cancer cell lines when compared to their parent molecules, and compound 16 a was more cytotoxic than compound **16b**. Hence, Chen et al. recommended further studies for compound **16a** [38].

**Figure 12:**
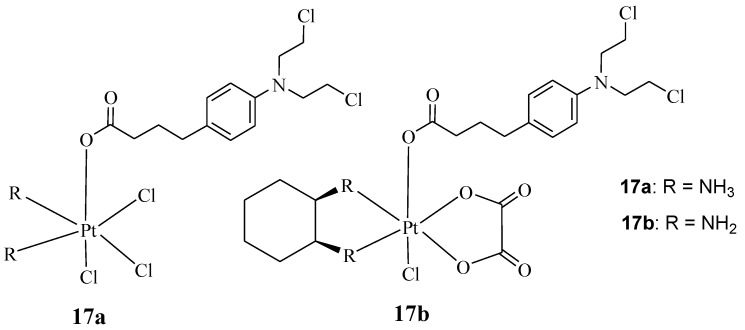
Chemical structures of platinum–Chlorambucil compounds **17a**,**b** synthesized by Qin et al. [39].

Qin et al. synthesized hybrids **17a**&**b**, containing Chlorambucil with oxaliplatin and cisplatin (Figure 12) [39]. Several human cancer cell lines, including breast, colorectal, gastric, and hepatocellular cancer cell lines, and cisplatin-resistant cell lines, were used to evaluate the synthesized drugs in vitro. The parent drugs (cisplatin, oxaliplatin, and chlorambucil) were used as controls [39]. The reported findings showed that against the drug-sensitive strains, compounds **17a** and **17b** exhibited improved cytotoxicity, with IC_50_ values in the range of 2.97–4.97 µM and 4.17–18.65 µM, respectively, when compared to Chlorambucil, with IC_50_ values in the range of 53.47–97.56 µM. However, the results were comparable to cisplatin and oxaliplatin [39]. Compound **17a** displayed remarkable anticancer activity compared to its counterpart and the parent drugs on the drug-resistant strain (SGC7901/CDDP) [39]. It was twice as active as cisplatin against SGC7901/CDDP, indicating its capability to overcome cisplatin drug resistance with a resistance factor of 1.42 compared to 3.35 for cisplatin [39]. Thus, there is a need for further studies to fully understand its mode of action. Moreover, it exhibited a 91.1% apoptosis rate, which was higher than Chlorambucil (5.2%) and cisplatin (83.6%) combined against SGC7901 cancer cell lines; a similar trend was observed against SGC7901/CDDP [39]. Therefore, hybridizing Chlorambucil with cisplatin offers significant potential to overcome drug resistance.

### 5.6. Chlorambucil Hybridized with Long-Chained Hydrocarbons and Fluorocarbon Chains

**Figure 13:**
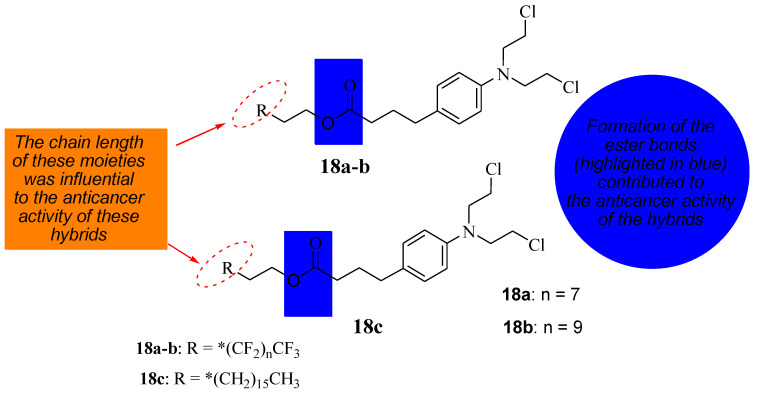
Chemical structure of Chlorambucil incorporated with hydrocarbon and fluorocarbon derivatives, the * represents the position where the R will bond (**18a**–**c**) [40].

To reduce the severe side effects associated with Chlorambucil, Nowak-Sliwinska et al. synthesized hybrid compounds (**18a**&**c**) using long hydrocarbon and fluorocarbon chains to inhibit tumor growth by altering Chlorambucil’s mode of action and improving its drug specificity (Figure 13) [40]. These compounds were evaluated against different human cancer cell lines in vitro and in vivo [40]. According to Clavel et al., Chlorambucil and compound **18a** displayed promising cytotoxic activity with IC_50_ values of 12 to 43 μM at 37 to 41.5 °C against A2780 and A2780 cisR cells (human ovarian carcinoma, cisplatin-sensitive and -resistant) [40,41]. The antitumor activity of compounds **18b** and **18c** was evaluated against human ovarian A2780 cancer cells in vivo [40]. The findings showed that, although the results are comparable to Chlorambucil, these two compounds can inhibit tumor growth through an anti-antiangiogenic effect. Additionally, compound **18c** with a long hydrocarbon chain was more active than 18b with a fluorocarbon chain, and it inhibited tumor growth at lower doses than **18b** in vivo [40]. The modification of the carboxylic group of Chlorambucil through the introduction of fluorocarbon and hydrocarbon long chains can alter the mechanism of action of Chlorambucil, resulting in improved anticancer outcomes. Modifying available clinically approved drugs is a promising approach to producing chemotherapeutics with enhanced selectivity and minimized side effects [40,41].

### 5.7. Chlorambucil–Asparagine Hybrid

**Figure 14:**
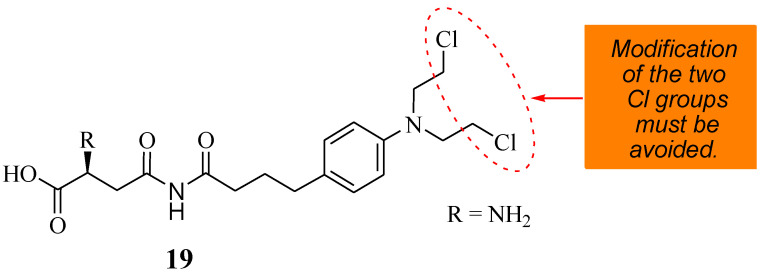
A novel therapeutic agent (**19**) chemical structure synthesized from Chlorambucil and asparagine moieties [42].

A novel therapeutic agent (**19**) (Figure 14) was prepared by Shafiee Ardestani et al. via a combination of Chlorambucil and asparagine to improve the anticancer effect of Chlorambucil [42]. Asparagine was used in the research due to the amino acid carrier’s important role in cancer cell growth and proliferation [42]. The anticancer effect of compound **19** was evaluated against the HT1080 cancerous cell line, and Chlorambucil was also evaluated to understand its effect on blood clotting and hemolysis [42]. Compound **19** was more cytotoxic, with an EC_50_ value of 81.8702 µM, than Chlorambucil, with an EC_50_ value of 138.854 µM. The compound was effective even at low concentrations, and low doses of the drug reduced the side effects. Compound **19** was not toxic to normal cell lines [42]. The mode of action of compound **19** was via apoptosis and did not influence the blood hemolysis rate and clotting factor. Hence, it is considered a promising anticancer drug that requires further studies [42].

### 5.8. Chlorambucil–Lipid Hybrids

**Figure 15:**
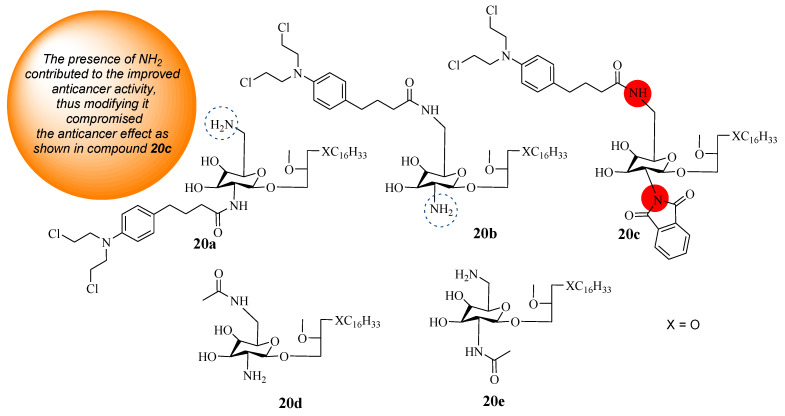
Chemical structures of Chlorambucil–lipid hybrids (**20a**–**c**) and modified lipids (**20d** and **20e**) [43].

Three hybrid compounds (**20a**–**c**) were synthesized using known synthetic protocols by Idowu et al. through the combination of Chlorambucil and glycosamine-derived glycerolipids (GDGs) (Figure 15) [43]. These compounds were evaluated against different prostate, breast, and pancreas cancer cell lines in vitro. Substituents were introduced to positions (C-2* and C-6*) of the GDG moiety to investigate the SAR [43]. Idowu et al. also considered each parent molecule’s effect on the synthesized hybrids’ anticancer activity. Thus, GDG derivatives (**20d** and **20e**) were synthesized and used in the study as references [43]. The findings in Table 3 showed that the hybrid molecules **20a**–**c** were selective towards cancer cell lines. Compounds **20a** and **20b** were the most active anticancer hybrids compared to the parent drug (CC_50_ > 150 µM) against all cancer cell lines except the pancreas cancer cell line. Compound **20c’s** CC_50_ values were greater than 20 µM, and it did not induce any significant activity against all cancer cell lines [43]. The presence of NH_2_ in the glucose moiety influences the anticancer activity of these hybrids and should not be modified [43]. Furthermore, for hybrids **20a** and **20b**, the position (*C-2* or *C-6*) of NH_2_ in the glucose moiety to which Chlorambucil was introduced displayed no significant impact on the anticancer activity of these hybrid drugs [43]. In contrast, hybrid **20a** exhibited a CC_50_ equal to 6.0 µM while **20b** exhibited a CC_50_ equal to 7.5 µM against prostate (DU145) and breast (JIMT1) cancerous cells, respectively. However, these hybrids displayed no significant effect against the pancreas (MiaPaCa2) cancer cell line [43]. Compounds **20d** and **20e** (CC_50_ > 15 µM) were less active than compounds **20a**&**b** except on the MiaPaCa2 cancer cell line, revealing that Chlorambucil contributed to the anticancer effect of the hybrids. The cytotoxicities of hybrids **20a** and **20b** were comparable to those of glycosamine-derived glycerolipids, indicating that further research is needed to investigate the mechanism of action of these compounds [43].

### 5.9. Chlorambucil Hybridized with DNA/ HDAC Inhibitors

**Figure 16:**
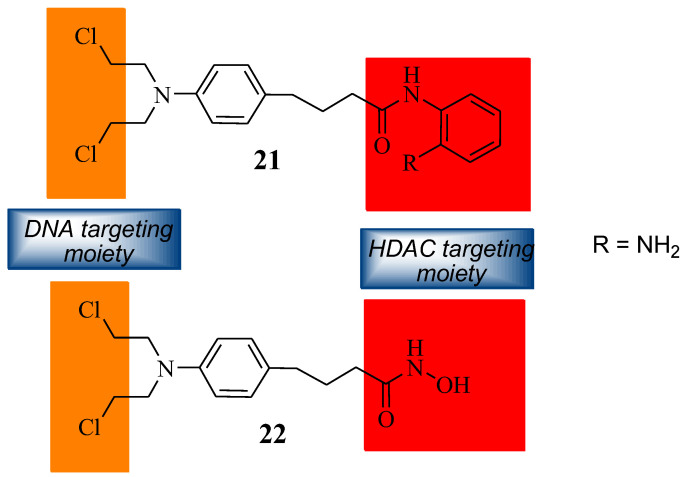
Chemical structures of Chlorambucil–DNA-HDAC inhibitors (**21** and **22**) [44,45].

The synthetic modification of Chlorambucil to improve its anticancer activity was continued by Xie et al. through the synthesis of a Chlorambucil–tacedinaline hybrid molecule **21** (Figure 16). The anticancer activity of the compound was tested against selected cancer cell lines, H460, A549, HepG2, SMMC77212, A375, and H1299 [44]. Compound **21** exhibited better anticancer activity (IC_50_ values 3.1–14.2 µM) than Chlorambucil (IC_50_ values 22.2–163.0 µM) and tacedinaline (IC_50_ values 11.3–33.0 µM) against all the cancer cell lines. The IC_50_ values of compound **21** were 1.3–6.1-fold and 3.6–40.8-fold lower than tacedinaline and Chlorambucil, respectively [44]. The compound acted as a dual-targeting molecule HDAC/DNA inhibitor [44].

Song et al. prepared Chlorambucil–hydroxamic acid hybrid compound **22** (Figure 16) [45]. The synthesized hybrid compound **22** was evaluated for its anticancer effect against several human cancer cell lines, including two breast cancer cell lines (MCF-7 and MDA-MB- 231), two leukaemia cell lines (U-937 and HL-60), and one ovarian (A2780) cancer cell line [45]. It exhibited superior antiproliferative activity compared to Chlorambucil against the human leukaemia cancer cells. In contrast, the synthesized compound exhibited poor antiproliferative activity against other cancer cell lines [45]. This compound displayed great activity with GI_50_ values of 1.24 and 1.75 µM against HL-60 and U-937 compared to 21.1 and 37.7 µM of Chlorambucil, respectively [45]. Hybrid **22** was not toxic to normal cells. Therefore, it can be a better agent for the treatment of cancer [45].

**Figure 17:**
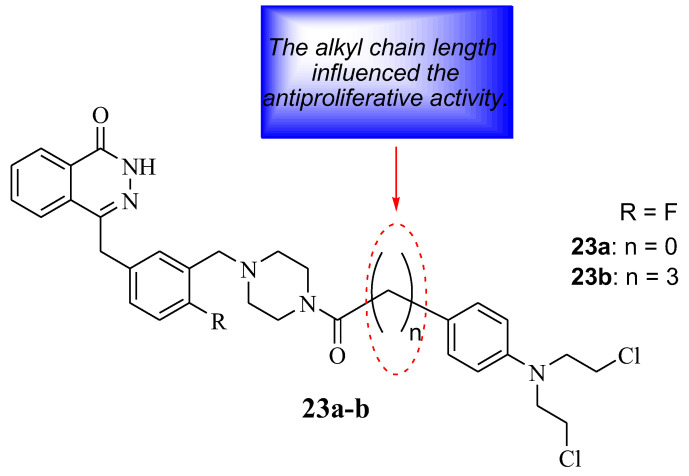
Chemical structure of Chlorambucil–olaparib hybrid compounds **23a**,**b** [46].

Qin et al. developed Chlorambucil–olaparib hybrids **23a**,**b** as potential anticancer compounds (Figure 17). They were evaluated for their anticancer effect against several human breast cancer cell lines in vitro. Hybrid **23** a displayed better antiproliferative activity towards some cancer cell lines, whereas hybrid **23** b exhibited superior antiproliferative activity against all selected cancer cells (except A549) compared to Chlorambucil (IC_50_ values between 1–100 µM) and olaparib (IC_50_ values 0.47–66.8 µM), with IC_50_ values ranging between 0.18–14.1 µM and 0.13–11.7 µM, respectively [46]. Noteworthy, the length of the linker between the two moieties influenced the antiproliferative activity of these compounds. Thus, compound **23b** was submitted for further analysis. The apoptotic analysis was consistent with the antiproliferative findings as hybrid **23b** displayed a prominent apoptotic rate at low doses of 1 and 2 µM compared to the parent drugs at high doses (5 and 10 µM) (Table 4) [46]. Therefore, hybrid **23b** can be a potential anticancer agent, but further optimization is essential [46]

### 5.10. Chlorambucil–Triphenylphosphonium Hybrids

**Figure 18:**
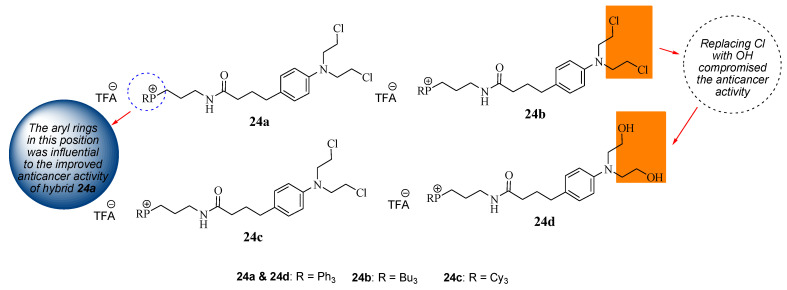
Chemical structure of mitochondrial–targeting Chlorambucil hybrids **24a**–**d** [47].

Millard et al. repurposed Chlorambucil by modifying it with the triphenylphosphonium group to obtain hybrid compounds **24a**–**c** via the amidation reaction between the carboxylic group of Chlorambucil and the amine group of phosphonium salt derivatives (Figure 18) [47]. These compounds targeted the mitochondria of the tumor cell as cancer cells have higher intrinsic mitochondrial membranes than normal cells. Additionally, compound **24a** halogens (Cl) of the Chlorambucil moiety were converted to hydroxyl groups, resulting in compound **24d**. They were evaluated against several cancer cell lines (breast and pancreatic cancer cell lines) in vitro [47].

The in vitro results revealed that although all the hybrids displayed superior anticancer activity compared to Chlorambucil against MCF7 breast cancer cell lines, compound **24a** exhibited remarkable anticancer activity with an IC_50_ value of 7.0 µM compared to other hybrid drugs with IC_50_ values between 35.0 and 80.0 µM against MCF7. The aryl rings contributed more to the cytotoxic effect of the molecules than saturated hydrocarbons. However, **24d** exhibited inferior activity compared to its derivative **24a**, suggesting that the modification of the chloro groups reduced the anticancer effect of the molecule [47]. Compound **24a** was further analyzed against breast and pancreatic cancer cell lines to validate the MCF7 results [47]. Against BxPC-3 and MIAPaCa-2 (pancreatic) cancer cells, compound **24a** displayed a significant increase in anticancer activity with IC_50_ values of 2.5 and 1.6 µM, respectively, which was more potent than Chlorambucil. Compound **24a** displayed superior activity on all the breast cancer cell lines compared to Chlorambucil, with IC_50_ values between 1.7 and 9.5 µM [47]. The mode of action of compound **24a** was more dominant than that of Chlorambucil. There is a need for more research on the development of mitochondrial-DNA-targeting therapeutics.

### 5.11. Chlorambucil–Honokiol Hybrid

**Figure 19:**
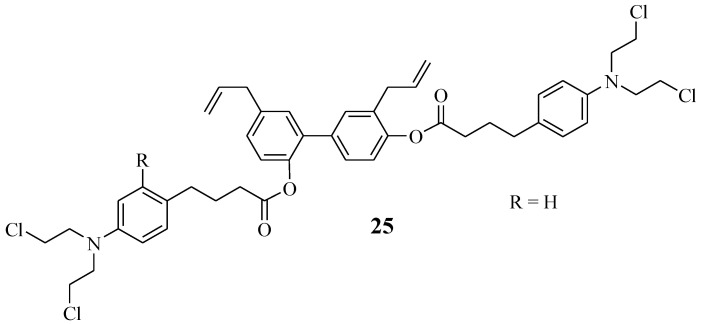
Chlorambucil–honokiol hybrid chemical structure **25** [51].

Honokiol is extracted from natural products, such as the tree leaves, bark, and seed cones of Magnolia officinal. It acts by targeting the mitochondria of the cancer cells and preventing metastasis [48,49,50,51]. Hence, Xia et al. combined it with Chlorambucil via ester linkage, resulting in hybrid compound **25** with an improved anticancer effect (Figure 19) [51]. Xia et al. biologically evaluated compound **25** against several human leukemic cell lines, U937, CCRF-CEM, MV4–11, Jurkat, and K562, in vitro [51]. That study revealed that compound **25** exhibited higher antiproliferative activity than its parent drugs, Chlorambucil and honokiol, against human leukemic cell lines with IC_50_ values 1.09–4.86 µM, 6.73–25.90 µM, and 10.60–23.76 µM, respectively [51]. Moreover, this compound displayed no cytotoxic effect against normal cancer cell lines in vitro. In vivo results indicated that this hybrid showed no physiological toxicity and inhibited leukaemia cell growth. Thus, this compound displayed a better therapeutic effect than Chlorambucil in vitro and in vivo [51].

### 5.12. Chlorambucil–Polyamide Hybrids

**Figure 20:**
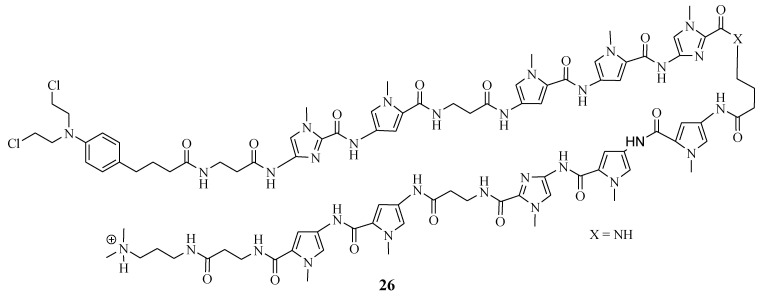
Chemical structure of Chlorambucil–polyamide hybrid **26** synthesized by Funakoshi et al. [52].

**Figure 21:**
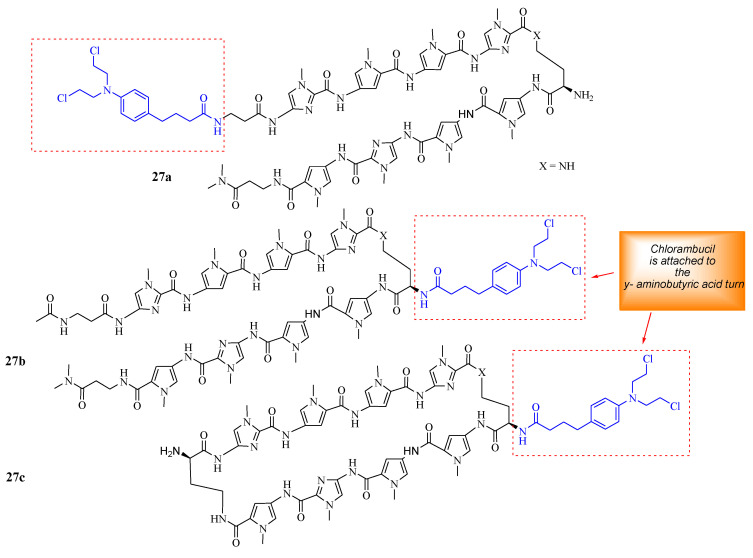
Chemical structures of Chlorambucil–polyamide hybrids **27a**–**c** synthesized by Hirose et al. [53].

Funakoshi et al. synthesized Chlorambucil–polyamide hybrid **26** and evaluated its antiproliferative effect against cancerous prostate cells in vitro (Figure 20) [52]. The in vitro results indicated that compound **26** was more effective than Chlorambucil and polyamide, with IC_50_ values between 0.984–4.643 µM, 160.3–821.3 µM, and 22.21–47.88 µM, respectively [52]. Notably, the in vivo studies demonstrated that compound **26** exhibited enhanced tumor inhibition growth without notably severe side effects when evaluated using 22 Rv1 xenografts. Therefore, further in vivo studies are recommended for this novel therapeutic agent [52]. Hirose et al. combined Chlorambucil with cyclic polyamides to develop novel hybrids **27a**–**c** (Figure 21) for cancer treatment [53]. After synthesizing compounds **27a**–c, the alkylation activity of the synthesized compounds against the human prostate cancer cell line (LNCaP) in vitro was studied. The cytotoxicity results showed that compound **27b** (IC_50_ = 0.074 µM) had a better/comparable cytotoxic effect compared to **27c** (IC_50_ = 0.093 µM) and compound **27a** (IC_50_ = 0.60 µM), respectively. However, **27c** had better alkylating activity than **27b**. Therefore, compound **27c** was overall the most active anticancer compound compared to **27a** and **27b**. Notably, SAR displayed that the attaching position of Chlorambucil into the polyamides could be influential to the anticancer activity of these novel hybrid compounds, as in compound **27a** (least active compound), it was attached on the *N*-terminus versus the y-aminobutyric acid turn on compounds **27b** and **27c**. Hence, the overall results indicated that **27c** is a novel DNA-alkylating chemotherapeutic agent, and further studies are recommended [53].

### 5.13. Chlorambucil–Phenosafranin Hybrid

**Figure 22:**
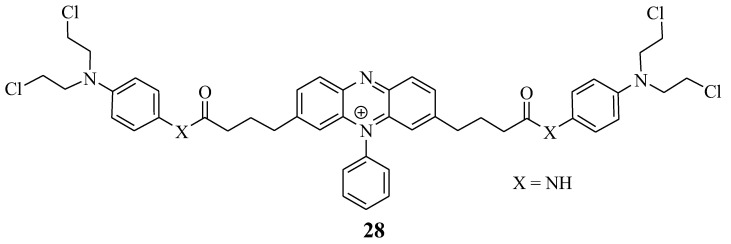
Chemical structure of Chlorambucil–phenosafranin hybrid **28** [54].

Miksa et al. developed a new hybrid drug, **28**, to treat cancer by combining Chlorambucil and phenosafranin through amide bonds (Figure 22) [54]. This drug’s anticancer activity was tested on HeLa cancer cell lines in vitro. Compound **28** exhibited improved anticancer activity, indicating that this is a promising chemotherapeutic agent. However, in vivo studies are needed to establish this anticancer agent’s biodistribution [54]. Overall, this hybrid drug is a promising chemotherapeutic agent.

### 5.14. Chlorambucil–Artemisinin Hybrids

**Figure 23:**
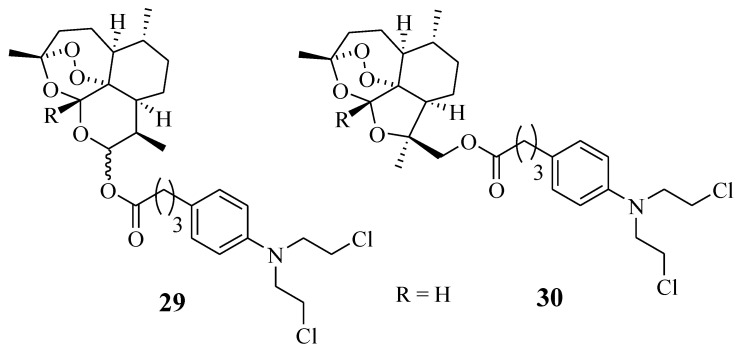
Chemical structure of Chlorambucil–artemisinin hybrid **29**–**30** synthesized by Dai et al. [55].

Chlorambucil derivatives **29**–**30** were among the sixteen artemisinin-nitrogen mustard anticancer agents synthesized by Dai et al. (Figure 23) [55]. These compounds were evaluated for their cytotoxic effect against several leukaemia cancer cell lines in vitro [55]. Compound **29** was less effective than the parent drugs, dihydroartemisinin and Chlorambucil, in vitro. In addition, compound **30** was selective against the cancer cell lines and displayed improved anticancer effect against HCT-116, A549, and CCRF-CEM with IC_50_ values of 12.333 ± 0.647 µM, 14.878 ± 0.844 µM, and 1.38 ± 0.042 µM, respectively, in comparison with the parent molecules [55]. However, further in vivo study to validate the results is crucial.

### 5.15. Chlorambucil–Evodiamine Hybrids

**Figure 24:**
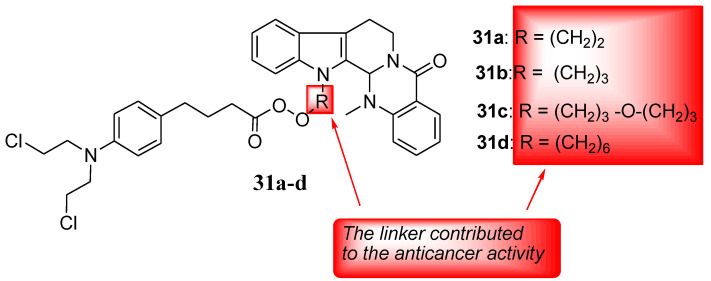
Chemical structures of Chlorambucil–evodiamine hybrid drugs **31a**–**d** synthesized by Hu et al. [60].

Evodiamine is a quinolone extracted from Evodiae fructus (Chinese herb), and it exhibits myriad biological activities, such as antitumor, anti-inflammatory, and anti-Alzheimer properties [56,57,58,59]. Thus, Hu et al. hybridized it with nitrogen-mustard and used four human cancer cells (HL-60, PC-3, THP-1, and HepG2) to evaluate a series of novel hybrids’ (including Chlorambucil) anticancer activity. The synthesized compounds were also tested against normal human cells (PBMC) in vitro [60]. Against all the cancer cells, Chlorambucil–evodiamine derivatives **31a**–**d** exhibited lower antiproliferative activity than the parent drugs (Figure 24). In contrast, against HL-60 cancer cells, compound **31c** exhibited more significant anticancer activity than the parent drugs, with an IC_50_ value of 1.29 µM in vitro [60]. Therefore, combining nitrogen-mustard derivatives and evodiamine is a promising approach to developing potent chemotherapeutics. The type of the linker and the length influenced the anticancer activity of the synthesized derivatives, as compounds with (CH_2_)_3_-O-(CH_2_)_3_ and (CH_2_)_3_ between the two parent moieties exhibited stronger activity than compounds with (CH_2_)_6_ and (CH_2_)_2_ between the moieties [60]. Therefore, the nature and length of linkers must be considered when developing hybrid compounds.

### 5.16. Chlorambucil–Brefeldin Hybrids

**Figure 25:**
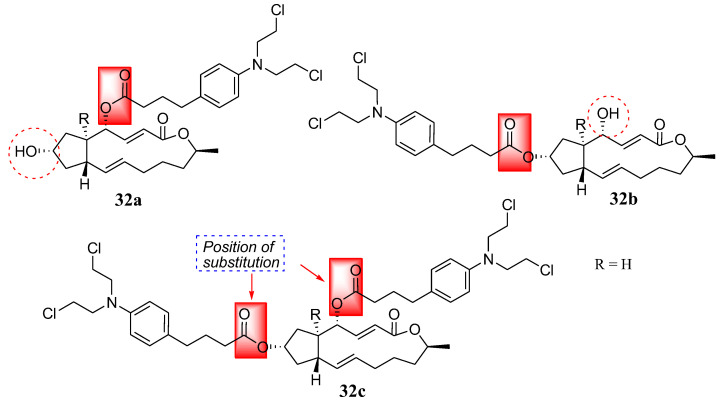
Chemical structure of Chlorambucil–brefeldin hybrids **32a**–**c** synthesized by Han et al. [61].

Another natural product, brefeldin, was coupled with Chlorambucil by Han et al. in a study of nitrogen-mustard derivatives, and compounds **32a**–**c** were obtained (Figure 25) [61]. Brefeldin is extracted from Penicillium decumbens, displaying several biological activities such as antiviral, antifungal, anticancer, and antimitotic properties [61,62,63,64]. The synthesized compounds were evaluated in vitro against multidrug-resistant cancer cells (Bel-7402/5-FU) and three human cancer cell lines (Bel-7402, HL-60, and PC-3) [61]. The compounds were selective against cancer cell lines and multidrug-resistant strains in vitro. Compounds **32a** (IC_50_ = 1.93 and 1.37 µM) and **32b** (IC_50_ = 4.86 and 6.84 µM) exhibited remarkable cytotoxicity activity against Bel-7402 cells and PC-3, respectively. Additionally, **32c** (IC_50_ = 7.25 µM) showed significant antitumor activity against Bel-7402 [61]. Furthermore, against Bel-7402/5-FU, all hybrids displayed significant activity, with IC_50_ ranging from 8.35 to 15.63 µM. Overall, all the hybrids exhibited higher antitumor activity than the parent molecules in vitro. Compounds substituted at the hydroxyl group at position 4 (**32a**) exhibited stronger antitumor activity than compounds substituted at the hydroxyl group at positions 7 (**32b**) and 4, 7 (**32c**). Therefore, the substitution position is essential and can influence the cytotoxicity of the molecules [61].

## 6. Future Perspectives and Conclusions

Many researchers have indicated that the number of cases of cancer may increase significantly in the next few years. The currently available anticancer drugs suffer from limitations, such as toxicity and multidrug resistance. The slow progress in discovering and developing new and effective chemotherapeutics is further hampering effective cancer treatment. Chlorambucil is an anticancer drug that is limited by a lack of specificity. Several hybrid drugs have been developed to minimize the limitations mentioned above.

**Figure 26:**
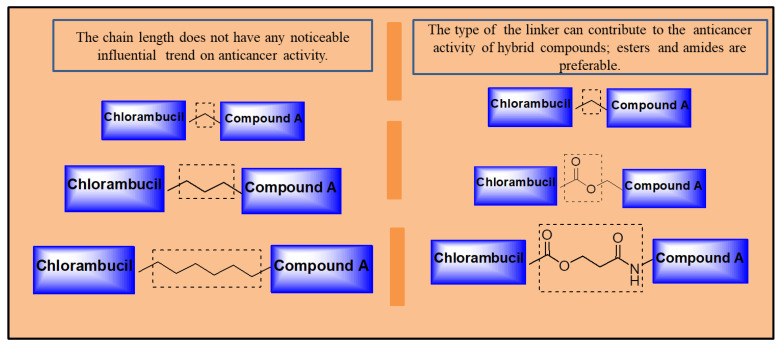
Possible linkers to develop hybrid drugs.

Several strategies have been reported and found to be effective in improving chlorambucil’s anticancer activity (Figure 26). Hybrid drugs are promising agents to treat cancer, as most Chlorambucil hybrid compounds reported in this review displayed improved biological activity. However, the hybrids were selective towards human cancer cell lines. Therefore, evaluating the hybrid derivatives on several cancer cell lines is recommended. Notably, to synthesize Chlorambucil hybrid drugs, the position of modification, type of linker, and functional groups must be considered, as these features influence the anticancer activity of the hybrids. One important factor is that the chain length of the linker between Chlorambucil and some pharmacophores did not induce a significant trend in the cytotoxic effects of the hybrid molecules. However, in some cases, the type of linker between Chlorambucil and other moieties influenced the anticancer activity of hybrid compounds. Thus, cleavable linkers such as esters and amide bonds are preferable (shown in Figure 26).

**Figure 27:**
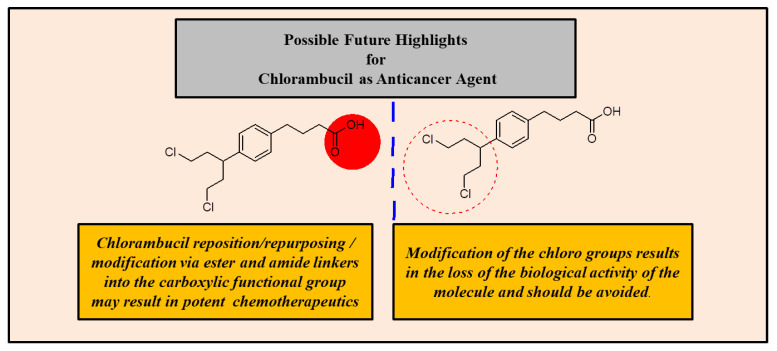
Possible solutions to improve Chlorambucil’s efficacy.

The modification of Chlorambucil’s di-chloro moiety is not recommended because it is responsible for the anticancer activity of the drug. Thus, modifying the moiety mostly resulted in hybrid compounds with poor anticancer activity. On the other hand, hybrid compounds combined using the carboxylic moiety of Chlorambucil resulted in improved and effective hybrid compounds with the formation of ester and amide bonds which are biologically friendly and easily cleaved in an enzymatic environment (demonstrated in Figure 27). Furthermore, the synthetic route used for the modification of Chlorambucil also affected the yield of the product. For instance, using synthetic modification routes consisting of steps is not recommended as they result in low yields as compared to routes with fewer steps. Additionally, nanotechnology is one promising alternative to improve drug transport and overcome the pharmacological limitations of conventional drugs.

**Figure 28:**
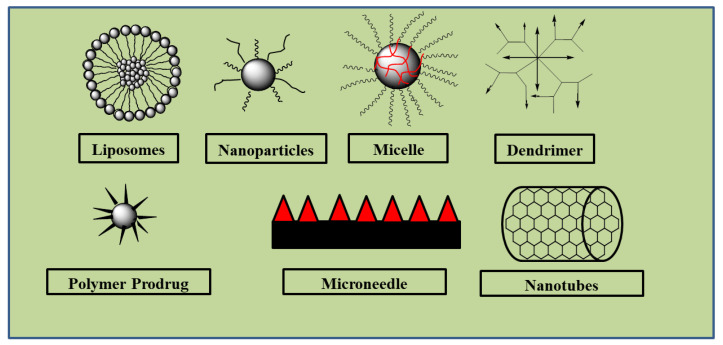
Examples of nanocarriers.

Chlorambucil is associated with challenges such as bioavailability and severe side effects on normal tissues. The incorporation of Chlorambucil-based hybrid molecules into nanocarriers, such as liposomes, dendrimers, micelles, etc., can improve their uptake and therapeutic outcomes with the potential to result in potent anticancer agents. Most of the Chlorambucil hybrid drugs were evaluated in vitro. In vivo studies are needed to validate the findings obtained from the in vitro analyses. The mode of action of these hybrid compounds is not fully understood. The highlighted limitations of Chlorambucil include poor cellular uptake, resulting in poor specificity and toxic effects on healthy tissues and organs. Therefore, it is important to study the introduction of these hybrid molecules into nanocarriers to further improve their drug biodistribution, pharmacokinetics, and stability properties (See Figure 28). There is no doubt that continuous studies of Chlorambucil-based hybrid compounds will result in potent chemotherapeutic agents.

## Figures and Tables

**Table 1:** The IC_50_ (µmol/L) values of the synthesized compounds and their parent compounds tested against PC3 and A2780/CP70 cancer cell lines.

Compound	Cell Lines	
	PC3	A2780/CP70
T-Platin-Chlorambucil–NPs	0.22 ± 0.04	1.0 ± 0.50
NT-Platin-Chlorambucil–NPs	0.46 ± 0.42	1.30 ± 0.02
Hybrid 14	0.60 ± 0.06	5.2 ± 0.80
2Chlorambucil + Cisplatin	14.00 ± 5.00	12.00 ± 5.00
Cisplatin	13.50 ± 2.00	13.50 ± 4.00
Chlorambucil	181.00 ± 15.00	200.00 ± 40.00

**Table 2:** The in vitro cytotoxicity results of compound **15** and the parent drugs [IC_50_ (µM) ± SD] [37].

Cancer Cell Lines	Compound		
	15	Platinum	Chlorambucil
BCPAP	0.51 ± 0.20	7.31 ± 1.20	>100
HCT-15	0.39 ± 0.10	15.28 ± 2.60	44.52 ± 9.50
BxPC3	1.74 ± 0.50	7.22 ± 2.20	>100
A431	0.41 ± 0.10	2.21 ± 0.40	74.48 ± 8.2 > 100
LoVo	0.98 ± 0.10	9.15 ± 2.10	>100
C13	0.28 ± 0.10	22.11 ± 3.20	>100
2008	0.31 ± 0.10	2.18 ± 0.90	34.58 ± 16.50
PSN1	0.42 ± 0.10	18.11 ± 3.20	>100

**Table 3:** In vitro cytotoxicity results of compounds **20a**, **20b**, **20d**, and their parent drug (GDG) [CC_50_ (µM)] [43].

Cancer Cell Lines	Compound			
	GDG	20a	20b	20d
PC3	13.5	12.0	11.5	14.5
DU145	10.0	6.0	11.0	15.0
BT474	Not Tested	12.5	12.5	13.5
MDA-MB-231	7.1	8.5	10.5	13.5
JIMT1	9.0	8.5	7.5	11.0
MiaPaCa2	9.0	16.0	20.0	10.0

**Table 4:** Apoptotic rate (%) of hybrid compound **23b** and the parental drugs (olaparib and Chlorambucil) at different doses (µM) [46].

Compound	Dose (µM)	Apoptotic Rate (%)
23	1 & 2	10.96 & 12.69
olaparib	5 & 10	1.53 & 1.91
chlorambucil	5 & 10	2.49 & 2.96

## Data Availability

Not applicable.
